# A Different Perspective for Management of Diabetes Mellitus: Controlling Viral Liver Diseases

**DOI:** 10.1155/2017/5625371

**Published:** 2017-03-02

**Authors:** Yingying Zhao, Huichun Xing

**Affiliations:** Department of Hepatology, Division 3, Beijing Ditan Hospital, Capital Medical University and Teaching Hospital of Peking University, 8 Jingshundong Street, Beijing 100015, China

## Abstract

Knowing how to prevent and treat diabetes mellitus (DM) earlier is essential to improving outcomes. Through participating in synthesis and catabolism of glycogen, the liver helps to regulate glucose homeostasis. Viral related liver diseases are associated with glycometabolism disorders, which means effective management of viral liver diseases may be a therapeutic strategy for DM. The present article reviews the correlation between DM and liver diseases to give an update of the management of DM rooted by viral liver diseases.

## 1. Introduction

Insulin deficiency and/or insulin resistance, which causes glycometabolism disorders, leads to the symptoms of diabetes mellitus (DM). DM is an increasingly recognized global health concern. By 2030, the prevalence of diabetes among adults is expected to rise from 6.4% to 7.7% worldwide. During the next decade, the number of adults with diabetes is expected to rise by 20% in developed nations and 69% in developing nations [1, 2]. Long-term complications of DM include micro- and macrovascular damage, which include dysfunction of eyes, kidneys, nerves, gastrointestinal tracts, hearts, and blood vessels. Serious illness or stress can result in acute metabolic disorders, such as diabetic ketoacidosis (DKA) and hyperglycemic hyperosmolar status. Recently, some studies suggested that diabetes heightens patients' susceptibility to several cancers, such as colorectal, pancreatic, liver, and kidney cancers [3]. Thus, DM and its long-term complications deteriorate the health of residents and cause many deaths, ultimately expending national resources. Diabetes prevention and early management, as well as proper treatment of DM-related complications, are especially important factors.

As is known so far, several pathogenic processes and different factors are involved in the development of diabetes, and the interactions among factors are complicated. Genetic, environmental, and immune factors all contribute to the development of DM. Obesity, hypertension, alcohol consumption, and tobacco smoking are all risk factors of DM. Meanwhile, insulin resistance and *β*-cell dysfunction are common mechanisms of DM, and glucose and lipid toxicities are important mechanisms of the development of DM [1, 2].

Glycometabolism disorder is the key point of the development of DM, and the regulation of glucose homeostasis is particularly important for maintaining normal glucose metabolism. The liver is an important organ that can regulate glucose homeostasis, by means of the synthesis and catabolism of glycogen. Once chronic liver damage onsets, glycometabolism disorders may follow. It is well documented that patients with liver diseases, such as hepatitis B, hepatitis C, nonalcoholic fatty liver disease (NAFLD), fatty liver, cirrhosis, and hepatocellular carcinoma (HCC), have high comorbidity of type 2 diabetes mellitus (T2DM) [4, 5]. Many studies have proposed that liver-injury patients are likely to have a greater risk of DM [6, 7]. Cirrhosis complications may also cause DM, leading to hepatogenous diabetes (HD) [6–9]. In contrast, prevention or treatment of viral hepatitis leads to a definite improvement of insulin sensitivity [10–12]. This review illuminates the underlying relationship between DM and viral liver diseases, as well as the possible mechanisms.

## 2. DM and Hepatitis B

### 2.1. Epidemiological Characteristics

Custro et al. [13] had discovered a relation between HBV and T2DM more than a decade ago. The following studies also proved this relationship [14, 15].

A meta-analysis by Cai and coworkers demonstrated that, among 15 studies analyzed, 6 presented studies found a greater rate of DM risk in HBV-infected patients than in noninfected subjects, 7 did not support this association, and 2 showed weak correlations. In the Asia-Pacific area, the rate of DM in patients with HBV was higher than in patients uninfected with HBV [odds ratio (OR) 1.67] [16]. Hepatitis B surface (HBsAg) carriers had a three times higher rate (32.9%) of developing gestational diabetes compared with the regular population [17]. The HBV-related cirrhotic patients with good liver function had relatively normal insulin levels [18]. There was a higher incidence of T2DM for CHB patients, and HBeAg status and HBV DNA levels affected this incidence [19]. A 10-year follow-up study suggested that HBV did not elevate the chances of developing T2DM, since asymptomatic HBV carriers did not have higher risks of developing T2DM than controls. The researchers proposed that, instead of the virus, perhaps HBV-related parenchymal liver deficiencies led to greater rates of DM [15, 20, 21].

### 2.2. Correlation and Mutual Influence

Papatheodoridis et al. found that DM risk was related to the severity of liver damage, the serum gammaglutamyl-transpeptidase (GGT) level, and the grade of fibrosis [20]. DM risk was not related to the etiology of liver diseases. Viral infection was not a risk factor of diabetes [15].

Patients with chronic hepatitis B (CHB) were more prone to developing hepatic steatosis than those without CHB [22]. Hepatic steatosis was prevalent among youthful males with ongoing CHB (51.2% of the patients), and triglyceride was the independent factor for hepatic steatosis, while steatosis and viral factors, including HBV DNA and hepatitis B e antigen (HBeAg) negative or positive, had no relationship. CHB was associated with IR [23]. Wang et al. also found that chronic HBV did not raise the likelihood of IR [24], while hepatitis patients of various etiologies had an increased incidence of diabetes. So their views were that the positive correlation of HBV and diabetes might be a result of HBV-induced liver damage instead of HBV [15]. Diabetes increases the risk of cirrhosis in CHB patients and promotes the progress of cirrhosis [25].

### 2.3. Mechanism

Many mechanisms may explain the relation of HBV and DM onset. The liver plays an important role in regulating glucose homeostasis. HBV-related liver damage and inflammation cause the disorder of glucose metabolism. Inflammation can reduce the effects of insulin on the liver, thus leading to liver dysfunction, which in turn induces IR [16, 26–29].

Serum insulin increases for patients with cirrhosis, since hepatic function disorders lead to abnormal insulin levels, which might decrease hepatic blood supply and inhibit insulin-stimulated glucose uptake [30]. The appearance of inducible nitric oxide synthase has been proved to exacerbate HBV-infection liver diseases. HBV is replicated in the extrahepatic tissues, such as the pancreas, which damages *β*-cells. HBsAg is detected in bile and pancreatic secretions [31], and the DNA of HBV is found in many extrahepatic tissues, such as the pancreas [32]. So accompanied by the injury of pancreatic *β*-cells, DM may occur after the disorder of glucose metabolism. Beyond that, IR may be involved in the development of hepatogenous diabetes. Ji et al. [33] claimed that pre-S2 protein downregulated insulin receptor genes, which results in IR [17]; then the level of serum soluble tumor necrosis factor receptors increases and participates in the regulation of gluconeogenesis [30].

Lin et al. found that immunization against hepatitis B could reduce the danger of developing diabetes by 33% (odds ratio 5 0.67, 95% confidence interval 0.52–0.84) [10]. In a cross-sectional study of 15,316 adult subjects, successful immunizations more positively correlated with a lower rate of diabetes (odd ratio (OR) 0.67, 95% CI: 0.52–0.84) [11].

## 3. DM and Hepatitis C

### 3.1. Epidemiological Characteristics

Since 1994, Allison et al. performed [34] epidemiological studies to claim the association between HCV, or HCV cirrhosis, and DM. It is generally considered that chronic HCV infection causes several extrahepatic complications, such as T2DM, IR, cognitive impairment, cardiovascular disorders (i.e., stroke, ischemic heart disease), and glomerulonephritis renal insufficiency [35, 36]. Viral eradication can change the clinical progression of patients with chronic hepatitis C (CHC) and T2DM [37–43]. More and more evidence shows the increased risk of T2DM in individuals with HCV infection [43, 44]. Much evidence shows that increasing severities of HCV are positively correlated with an increased risk of T2DM [43, 44]. The rate of DM in chronic HCV-seropositive populations in Europe, North America, and Asia ranged from 13 to 33% [45, 46]. Approximately 20–30% of CHC patients who had liver cirrhosis later developed diabetes. The incidence of DM was noticeably larger in people with HCV cirrhosis than from other etiologies, and HCV infection was detected prior to T2DM in most cases [46].

### 3.2. Correlation and Mutual Influence between the Two Diseases

 Hammerstad et al. reported that age, gender, family history, genotype of HCV, therapeutic regimen, and virological response were factors of T2DM in HCV patients. For example, the danger of developing diabetes was higher in people who had a positive family history than in those with only one diabetic parent, while genotype 1 and sustained viral response (SVR) reduce the development of DM [47]. Zornitzki et al. claim that CHC patients with interferon (INF) treatment had a greater (10–18-fold) danger of developing type 1 diabetes mellitus (T1DM) than the common population, with a median onset age of 43 (range: 24–66 years) in Caucasians and 52 (range: 45–63 years) in Japanese. Most patients developed T1DM during the treatment. The median time of onset was 4.2 months for Caucasians and 5.7 months for Japanese [48]. But in another study among the US population, the authors found that there was no distinction between the incidence of diabetes and prediabetes by HCV status, and HCV was not related to diabetes or to IR in people with normal glucose levels. In contrast, higher GGT and alanine aminotransferase (ALT) levels were related to diabetes independent of HCV infection [49].

With or without cirrhosis, DM reduces SVR [7]. In a different study, researchers claim that a mix of metformin, pegylated interferon, and ribavirin prolonged the SVR and increased insulin sensitivity only in women having CHC [47]. A few studies showed a significantly reduced incidence of T2DM among CHC patients with SVR [50, 51]. Arase et al. retrospectively analyzed a cohort of 2,842 patients with HCV who were treated with IFN monotherapy or both IFN and ribavirin. They found that the yearly rate of T2DM development in people with HCV was 0.8% to 1.0% and that SVR reduced the risk of T2DM onset by two-thirds in patients with HCV who accepted antiviral treatment [52]. Pavone et al. retrospectively evaluated 149 HCV-positive diabetics receiving direct antiviral drugs (DAA) and found that the subjects could gain a rapid reduction of fasting glucose (FG) levels [12]. Antiviral therapy, which eradicated HCV, decreased the rate of DM onset [53].

Imazeki et al. found that there was a greater rate of DM and IR in patients infected with HCV than in those infected with HBV [54]. Other studies did not validate the difference [21, 55, 56]. In addition to HCV, other risk factors that may lead to glucose abnormalities include age, gender, BMI, and cirrhosis [54].

### 3.3. Mechanisms

Through increasing oxidative stress, IR, and glucose intolerance, HCV results in hyperuricemia, arterial hypertension, and atherosclerosis, thus damaging the cardiovascular system [57]. HCV likely increases the risk of T2DM through elevating IR [58]. Studies have elucidated the mechanisms through which HCV impairs the insulin-signaling pathway in liver cells [59]. Specific steps include targeting the serine phosphorylation of insulin receptors (IRS), increasing levels of tumor necrosis factor-*α* (TNF-*α*), overexpressing inhibitors of cytokines (SOC-3), inducing SOC-7 [39], increasing reactive oxygen species 2 and other inflammatory cytokines3, and directly alternating insulin signaling by HCV4 and *β*-cell dysfunction [46].

## 4. DM and Viral Cirrhosis

### 4.1. Epidemiological Characteristics

Epidemiological data indicates that DM is associated with liver cirrhosis. Studies have found that many DM patients have a high rate of cirrhosis and advanced fibrosis [59]. Nearly 80% of people with liver cirrhosis also have problems metabolizing glucose, with 30% developing DM [60]. DM participates in the progress of liver diseases. Elkrief et al. retrospectively assessed the effect of DM on hospitalized patients with liver decompensation, liver transplantation, and mortality. They found that diabetes was an independent prognostic indicator which is unrelated to the model for end-stage liver disease (MELD) score. Diabetes significantly decreases transplantation-free survival [61].

Sporea et al. used Transient Elastography to evaluate stages of liver fibrosis in DM patients. They found that 18.8% of patients with DM had significant fibrosis and 13.8% had cirrhosis, rates which are significantly higher than in the common population [62].

Jepsen and colleagues found that 22% of the patients with cirrhosis and first-onset hepatic encephalopathy (HE) had diabetes. Child-Pugh class C patients with DM had higher risk for HE than those without diabetes [63].

### 4.2. Mechanisms

Inflammation and fibrosis are increased in DM, and DM elevates the hepatic complications and mortality hazard in patients with cirrhosis. The possible mechanism of liver damage is that adipokines increases mitochondrial oxidative stress which promotes inflammation and fibrosis [7]. In addition, inflamed subcutaneous and visceral adipose tissues produce proinflammatory factors such as tumor necrosis factor-*α*, leptin, interleukin 6, and adiponectin. Several of these factors signal stellate cells to produce more collagen, leading to a greater release of growth factors for connective tissue, which accumulates proteins in the extracellular matrix and results in fibrosis containing immuno-competence. This condition increases the danger of infections, including bacterial peritonitis, which may lead to high death rates [7, 64]. DM contributes to the activation of hepatic stellate cells, inflammation, apoptosis, angiogenesis, and hepatic sinusoidal capillarization, which progresses liver fibrosis and cirrhosis. Patients with cirrhosis have reduced liver insulin clearance and increased advanced glycation end-products, hypoxia, and hypoxia-inducible factors [63].

### 4.3. Correlation and Mutual Influence between the Two Diseases

DM treatment improves the survival rate of patients with liver cirrhosis. Metformin is related to a rise in survival rate and a reduction of liver complications [7]. The use of the sodium-glucose cotransporter-2 (SGLT2) inhibitors and incretin treatments, including oral inhibitors of dipeptidylpeptidase-4 (DPP-4) and injectable glucagon-like peptide-1 (GLP-1) receptor agonists, ameliorated diabetic conditions and reduced liver fibrosis and inflammation [60].

## 5. DM and HCC

### 5.1. Epidemiological Characteristics

The number of new onset HCC is 700,000 per year. HCC is the 3rd greatest cause of cancer-related deaths. Besides common risk factors, such as chronic viral hepatitis and liver cirrhosis, other hepatic diseases, including metabolic, autoimmune, and alcoholic liver diseases, may also elevate the risk of HCC [65, 66]. Studies of epidemiological data indicated that DM raises the danger of developing HCC. Factors included age, alcohol drinking, increased alkaline phosphatase levels, decreased serum triglyceride (TG) levels, increased GGT levels, increased aspartate aminotransferase-to-platelet ratio index (APRI) score, decreased platelet counts, and increased Fibrosis-4 (FIB-4) score. Multivariate Cox regression analysis claimed that age > 65 years, low TG levels (<150 mg/dl), and high GGT levels (>40 IU/L) were independent risk factors for HCC [67].

### 5.2. Correlation and Mutual Influence between the Two Diseases

Hung et al. found that DM was associated with both the rate of HCC and the survival rate. DM was associated with HCC progress among IFN-based antiviral therapy treated CHC patients [67, 68], especially the ones who obtained SVR, whereas, 2 years after acquiring SVR, the danger of progression to HCC may reduce [68]. Accepting IFN-based CHC patients with baseline DM having overall poor prognosis and lower survival rate than the non-DM patients (*p* < 0.001), a further analysis found that DM could act as an independent prognostic factor for HCC among noncirrhosis patients and also increase the likelihood of HCC onset of them [67].

Yang et al. confirmed the follow-up time related incidence of DM as 34% (253/739) and the rate of DM patients developing HCC after a follow-up of 38 months was 9% (69/739) by a study of 739 patients. Diabetes increased the danger of patients with non-HCV cirrhosis developing HCC. In HCV cirrhosis patients with already a high risk of HCC, diabetes might not elevate the risk any further [4]. Researchers also found that DM was an important risk factor for HCC among the CHC patients with SVR. In comparison to patients without DM, patients with cirrhosis and DM had a sevenfold higher risk for development of HCC, and the HCC risk per year for them was 7.9% during a two-year follow-up after SVR. As time went on, the risk declined [69].

Systemic risks of HCC, such as hyperinsulinemia, obesity-related hypoxia, systemic inflammation, systemic influences of cytokines and adipokines, systemic immune dysregulation, systemic effects of the gut microbiome, autophagy, and local factors all contributed to HCC risk [70].

### 5.3. Mechanisms

Hyperinsulinemia, which can induce tumor cell growth and metastasis in T2DM, is believed to cause carcinogenesis through affecting the proliferating pathway. The state targets the pathway, after insulin receptors, through the effect of insulin-like growth factor IGF-1 and is directly involved in carcinogenesis by acting on cancer cells. Insulin decreases the expression of IGF binding protein-1, thus increasing the bioactive IGF-1. In contrast to insulin, IGF-1 has more potent mitogenic and antiapoptotic effects, promoting growth of preneoplastic and neoplastic cells. Adiponectin, largely expressed by adipokines, has anti-inflammatory and antitumor roles. Adipose tissues can produce different kinds of inflammatory cytokines, such as interleukin-6, plasminogen activator inhibitor-1, and monocyte chemoattractant protein, which may aid the progression of cancer. Higher leptin and lower adiponectin levels could also increase the risk of cancer in patients with obesity or T2DM. The lasting action of inflammatory cytokines would interrupt the normal capacity for intracellular antioxidants, making cells more susceptible to malignant changes [71].

Hyperinsulinemia is believed to be an independent risk factor for HCC, and it is reported that major dysregulation of insulin dependent pathways was common in patients with HCC. Signals from IGF-I and more so from IGF-II affect the progression of HCC. Aberrant mammalian targets of rapamycin (mTOR) signaling in HCC have been suggested to exist in tumors. Additionally, DM is associated with elevated serum estrogen levels, which could reduce HCC progression by suppressing chronic low-grade hepatic inflammation. Recently, studies showed that the AMPK-independent pathway (represented by the LKB1/AMPK/mTOR axis), miRNAs downstream of this biguanide, and their messenger RNAs were the key points of cell survival and proliferation [72].

Some studies claimed that the condition of DM tended to progress HCC development in patients, in both the presence or the absence of cirrhosis [72]. Cytokines are important for both the mechanisms of IR and the glucose disposal defects, as well as the development of liver diseases. Capone et al. reported that the T2DM-HCC patients had higher levels of* ADIPOQ*, *β*-nerve growth factor (*β*-NGF), chemokine ligand1 (CXCL1), CXCL12, hepatocyte growth factor (HGF), several interleukin (IL) members, and IFN-*α* and lower levels of leptin than T2DM or HCC patients. They also had higher levels of CXCL9, platelet endothelial cell adhesion mole-1 (PECAM-1), prolactin, and glucagon and lower levels of soluble vascular endothelial growth factor sVEGFR-1 and sVEGFR-2 than T2DM. These patients had similar levels of CXCL9, PECAM-1, prolactin, glucagon, sVEGFR-1, and sVEGFR-2. The serum levels of TP53 in HCC and T2DM patients were higher and had no correlation with CXCL1, interleukin-2 receptor-alpha (IL-2R alpha), PECAM-1, and prolactin, whereas there was an important correlation between tumor protein p53(TP53) and CXCL12 in HCC and in T2D-HCC patients [73].

## 6. DM and Liver Transplant

### 6.1. Epidemiological Characteristics

Over recent years, increasing success rates of surgery and immunosuppressive treatments have led to high survival rates after liver transplantation (LT). Nevertheless, many transplant complications still reduce the survival rate, such as the development of de novo malignancies, recurrence of underlying diseases, obesity, hypertension, new-onset diabetes, dyslipidemia, and cardiovascular diseases [74]. A common complication is new-onset diabetes after transplantation (NODAT), which has an incidence of roughly 30% [75]. The specific rate of NODAT depends on different diagnostic criteria, the duration of follow-up, and the study populations. The rate of NODAT in China is similar to that in Western countries [76].

### 6.2. Correlation and Mutual Influence between the Two Diseases

Existing preoperative cirrhotic complications, such as ascites, esophageal varices, and hepatic coma, were risk factors for post-LT NODAT. In Western countries, the three major risk factors of NODAT are HCV infection, obesity, and alcoholic cirrhosis. The major risk factor in China is viral hepatitis [74–76].

### 6.3. Mechanisms

The early recurrence of hepatitis C and immunosuppressive drugs after liver transplantation is related to LT-NODA. Steroids increase insulin resistance and reduce *β*-cell secretion. Many recent studies show that intestinal microbiota took part in the regulation of carbohydrate metabolism and affected the pathogenesis of glucose metabolism disorders. It was noted that liver transplantation could affect intestinal microbiota through multiple factors, such as immunosuppression [74]. Glycaemic-controlled NODAT is also an independent risk factor of HCC recurrence. NODAT patients who received hypoglycaemic treatment had a worse prognosis and a higher HCC recurrence in comparison to those without treatment [76]. Actively intervening with these risk factors could decrease the occurrence rate of metabolic syndrome after liver transplantation and improve the patient's quality of life [77].

## 7. Summary

DM is constantly increasing around the world. Prevention and early treatment of DM improves the outcomes of DM patients. The relationships between DM and liver diseases are complex. The prevalence of DM in patients with liver diseases is higher than that in regular populations; the presence of diabetes is a predictor of worse outcomes in patients with liver diseases. In addition to healthy diet, regular physical activity, keeping a normal BMI, and avoiding smoking, the controlling of liver diseases, especially viral liver diseases, is also important to managing DM. Proper management of DM improves the outcomes of patients with liver diseases, HCC, and liver recipients.
